# Report of a Case of Monstrosity

**Published:** 1858-07

**Authors:** J. E. G. Terrill

**Affiliations:** Greenville, Georgia


### ARTICLE III.

*Report of a Case of Monstrosity.* By J. E. G. Terrill, M. D., of Greenville, Georgia.

Some two or three months since, I was called to see a negro woman, (the property of Jeremiah Frazier, of Meriwether County), said to be in labor. After an examination, I found it was not her full term. I prescribed accordingly and prevented the abortion, since which time she has been able to attend to her usual work in the farm, up to May 5th, about 11 o'clock, when she was taken in labour and progressed regularly with the exception of a slight hemorrhage, until 3 o'clock, P. M.; at which time she gave birth to a singularly developed child, a description of which I will endeavor to give as accurately as possible. I have it before me. The head is regularly formed, with quite a sufficiency of hair; likewise are the upper extremities and chest. The abdominal walls are wanting, with the exception of a small space above the pubes.—The liver and intestines are exposed fully to view, hanging loosely, and retained *in situ* by the reflections of the peritoneum. The lower extremities are reversed. They fold upon the back, instead of the abdomen. The anterior portion of the legs are the posterior portion in this case. The os calcis is in front, the toes pointing backwards. The great toes are upon the outside of the feet—the feet are flat, being made so doubtless from their position upon the occiput while in utero. It is strictly neuter gender—as it has no signs of the genitals of either male or female—the parts are perfectly smooth. The anus is above the os pubis, just below the margin that surrounds the viscera of the abdomen. The bladder is not visible. The spine is considerably distorted. Its weight is about seven pounds. It is hardly necessary to say it was still-born. The mother is doing well. I have thus given an outline of the monstrosity which I set out to do as near as possible, without going into particulars. I have not made any dissection of it at all, as I wish to preserve the specimen, and present it to the Museum of the Atlanta Medical College.

